# Unobtrusive Sensing Technology for Quantifying Stress and Well-Being Using Pulse, Speech, Body Motion, and Electrodermal Data in a Workplace Setting: Study Concept and Design

**DOI:** 10.3389/fpsyt.2021.611243

**Published:** 2021-04-28

**Authors:** Keisuke Izumi, Kazumichi Minato, Kiko Shiga, Tatsuki Sugio, Sayaka Hanashiro, Kelley Cortright, Shun Kudo, Takanori Fujita, Mitsuhiro Sado, Takashi Maeno, Toru Takebayashi, Masaru Mimura, Taishiro Kishimoto

**Affiliations:** ^1^Division of Rheumatology, Department of Internal Medicine, Keio University School of Medicine, Tokyo, Japan; ^2^National Hospital Organization Tokyo Medical Center, Tokyo, Japan; ^3^Medical AI Center, Keio University, Tokyo, Japan; ^4^Department of Neuropsychiatry, Keio University School of Medicine, Tokyo, Japan; ^5^Department of Health Policy and Management, Keio University School of Medicine, Tokyo, Japan; ^6^World Economic Forum Centre for the Fourth Industrial Revolution Japan, Tokyo, Japan; ^7^Center for Stress Research, Keio University, Tokyo, Japan; ^8^Human System Design Laboratory, Graduate School of System Design and Management, Keio University, Tokyo, Japan; ^9^Department of Preventive Medicine and Public Health, Keio University School of Medicine, Tokyo, Japan; ^10^Department of Psychiatry, Donald and Barbara Zucker School of Medicine, New York, NY, United States

**Keywords:** adult psychiatry, mental health, occupational & industrial medicine, wearabe sensors, well-being, stress, protocols, depression

## Abstract

**Introduction:** Mental disorders are a leading cause of disability worldwide. Depression has a significant impact in the field of occupational health because it is particularly prevalent during working age. On the other hand, there are a growing number of studies on the relationship between “well-being” and employee productivity. To promote healthy and productive workplaces, this study aims to develop a technique to quantify stress and well-being in a way that does not disturb the workplace.

**Methods and analysis:** This is a single-arm prospective observational study. The target population is adult (>20 years old) workers at companies that often engage in desk work; specifically, a person who sits in front of a computer for at least half their work hours. The following data will be collected: (a) participants' background characteristics; (b) participants' biological data during the 4-week observation period using sensing devices such as a camera built into the computer (pulse wave data extracted from the facial video images), a microphone built into their work computer (voice data), and a wristband-type wearable device (electrodermal activity data, body motion data, and body temperature); (c) stress, well-being, and depression rating scale assessment data. The analysis workflow is as follows: (1) primary analysis, comprised of using software to digitalize participants' vital information; (2) secondary analysis, comprised of examining the relationship between the quantified vital data from (1), stress, well-being, and depression; (3) tertiary analysis, comprised of generating machine learning algorithms to estimate stress, well-being, and degree of depression in relation to each set of vital data as well as multimodal vital data.

**Discussion:** This study will evaluate digital phenotype regarding stress and well-being of white-collar workers over a 4-week period using persistently obtainable biomarkers such as heart rate, acoustic characteristics, body motion, and electrodermal activity. Eventually, this study will lead to the development of a machine learning algorithm to determine people's optimal levels of stress and well-being.

**Ethics and dissemination:** Collected data and study results will be disseminated widely through conference presentations, journal publications, and/or mass media. The summarized results of our overall analysis will be supplied to participants.

**Registration:** UMIN000036814

## Introduction

Mental disorders are a leading cause of disability worldwide and, among mental disorders, major depressive disorder was ranked number 1 in years lived with disability in 2017 (1). The lifetime prevalence of depression in Japan is estimated at 6.2% (2002–2006 estimate), which makes it the country's most common mental illness (2). The disease costs are also enormous and are estimated to exceed 3.09 trillion Japanese Yen (~30 billion U.S. dollars) per year (3). Depression has a significant impact in the field of occupational health because it is particularly prevalent during working age (20–65 years of age). It is estimated that more than half of the social loss due to depression is attributed to loss of labor productivity through absenteeism and presenteeism (2). The Japanese government has implemented measures against long working hours (Standards on limits of overtime work in 1998; the revision of the Industrial Safety and Health Act in 2006) and has introduced the stress check system (enforced as of December 2015), but there has not been a significant impact from those measures. In fact, the number of workers' compensation claims related to mental disorders are increasing each year.

On the other hand, there are a growing number of studies on the relationship between “well-being” and employee productivity. The happiness of employees has been reported to be associated with creativity and productivity (4). As the birthrate continues to decline and the aging population continues to increase in Japan, the working age population is also decreasing, requiring each employee to make the most of his/her abilities. Therefore, preventing negative factors such as depression and promoting well-being are major challenges for health management in the workplace and ensuring a stable economy.

Conventionally, it is known that heart rate variability (HRV) reflects autonomic nerve activity and serves as an index of psychological and physical stress. There are many suggested indicators for stress, including standard deviation of all normal-to-normal R-R intervals (SDNN) and the percentage of successive R-R intervals that differ by >50 ms (pNN50) with time domain variables and low frequency (LF), high frequency (HF), and their ratio (LF/HF) as frequency domain variables (5).

In addition, techniques for estimating emotions and depressive symptoms have been developed based on the analysis of speech including formant frequencies (6). The autonomic nervous system and voice characteristics are closely related to each other because most of the vocal fold movement is stimulated by the recurrent nerve, which branches off from the vagus nerve. Johannes et al. reported that speech fundamental frequency increased with psychological load while there was no significant difference with physical load, suggesting that speech fundamental frequency is a good indicator of psychological stress (7). Nakatsu et al. reported that combination of linear prediction cepstral coefficients and pitch-related characteristics predicted classification of 8 emotions using artificial neural network (8).

Furthermore, electrodermal activity measured by a wearable device can reflect the activity of eccrine sweat glands that are controlled only by sympathetic nerve activity, and is therefore expected to be a stress indicator (9). Previous studies have used the above mentioned approaches to measure subjects' degree of stress; however, this prior research comprises only feasibility studies that have simply verified device performance, and/or studies with only a small number of patients or healthy individuals (10, 11).

Moreover, such approaches are only used to evaluate the so-called “short-term stress” of the study period, and are not necessarily reflective of the effects of medium- to long-term stress. In a workplace environment, it is expected that regardless of whether employees experience temporary stress, there should also be situations where people feel a sense of freedom and accomplishment when a task is completed or a problem overcome. To date, very few studies have revealed the relationship between vital data and mid- to long-term stress and well-being in the workplace (12).

With the development of information and communication technology, similar approaches trying to utilize such biological and/or behavioral data to identify depression are reported recently. For example, in the case of HRV, Dell'Acqua et al. reported that HRV reduction can be the predictor for depression as HRV of individuals with dysphoria and in those with past depression was lower than controls (13). Kemp et al. in their meta-analysis on HRV and antidepressant treatment, reported that depression was associated with reduced HRV, which decreased with increasing depression severity (14).

We have reported that the timing related speech features can reflect the severity of depression. Speech rate, pause time, and response time showed significant associations with the total score of Hamilton Depression Rating Scale (15, 16). We have also reported that body movement captured by infrared sensor can be reflective of depression severity (15, 17).

Not only using single modality but combining multimodal data and with machine learning approach it may be more realistic to screen depression or to predict severity of depression. Utilizing wrist band-type wearable device that record three-axis acceleration, heart rate, body temperature, and ultraviolet light exposure, we have reported that it was possible to identify patients with depression with an accuracy of 0.76, and to predict depression severity with a 0.61 correlation coefficient with Hamilton Depression Rating Scale score (15, 18).

This study, which is funded by the Japan Agency for Medical Research and Development (AMED), is an industry-academia collaborative research project that aims to develop new techniques for evaluating mid- to long-term stress and well-being using technologies that will not obstruct normal work environments. By doing so, we hope to promote healthy workplaces and, in the end, to prevent depression in the prime of life.

### Research Objectives

The general aim of this study is to develop a technique to quantify stress and well-being in a way that does not disturb the workplace. Our specific objectives are: (1) To evaluate the relationship between the obtained questionnaire-based stress and well-being scores and the employees' vital data, which are collected using: a technique for extracting pulse waves from an image captured by a camera attached to the employee's computer, a technique for extracting emotional components from speech, and a technique for measuring electrodermal activity using a wristband-type wearable device; and (2) to gather information regarding how and when employees are coping to reduce stress or promote enhanced well-being by comparing questionnaire-based stress and well-being scores and the employees' vital data.

## Methods and Analysis

### Study Design

This is a single-arm prospective observational study.

### Participant Criteria

#### Inclusion Criteria

Adult (>20 years old) workers at companies that often engage in desk work; specifically, a person who sits in front of a computer for at least half their work hours (3.5 h a day or more).

#### Exclusion Criteria

People who correspond to any of the following groups are excluded from this study:

(1) People currently receiving treatment for mental illness, such as depression;
(2) People who suffer from diseases that may affect the acquisition of biometric information. For example, those who have a disease or disorder that affects pulse wave data measurement (persons who have paralysis or involuntary movements on their faces, or heart disease), those who have a disease or disorder that affects speech data measurement (speech difficulty caused by vocal cord extraction, etc.), or those who have a disease or disorder that affects measurement with wearable devices (persons with paralysis of the extremities or involuntary movement, etc.);
(3) People who have difficulty operating a computer, such as using email or the internet;
(4) People who cannot offer biometric information to researchers due to business/security reasons.

### Participant and Public Involvement

This study was supported by AMED at the stage of developing proof of concept for quantification of stress and well-being using pulse, speech, body motion, and electrodermal data. The study design was made by industrial doctors who served as consultants for some of the companies for which the participants of this study work. These industrial doctors, who are members of our research team, conducted preliminary meetings with the participants, and based on those meetings, they arrived at the question this study hopes to answer: whether stress and well-being can be quantified by pulse, speech, and electrodermal data. The results of this study will be made available to participants through debriefing sessions at each participating company.

### Data Collection

Data will be collected according to the observation period schedule in Table 1.

**Table 1:** Schedule for data collection and evaluations during the study's observation period.

**Data collection**		**At the beginning**	**At the mid-point (2 weeks)**	**At the end (4 weeks)**
(A) Collection of background factors	Background characteristics (sex, age, department, work content, duration of service, etc.) Past stress check data, etc.	✓	If participant's environment changes, data will be updated.	
(B) Collection of data using external sensors	Pulse wave, speech, electrodermal activity, etc.	Acquired during business hours		
(C) Stress, well-being, and depression assessment using rating scale; self-reported daily condition	New occupational stress simple questionnaire (revised version): Estimated completion time, 5 min	✓	If participant's environment changes, data will be updated.	
	Perceived Stress Scale (PSS): Estimated completion time, 1 min			✓
	Satisfaction With Life Scale (SWLS): Estimated completion time, 1 min			✓
	Japanese version of Positive and Negative Affect Schedule (PANAS): estimated completion time, 1 min		✓	✓
	Japanese version of Flourishing Scale (FS-J): Estimated completion time, 1 min			✓
	Subjective well-being/ideal happiness: estimated completion time, 1 min			
	Japanese version of Patient Health Questionnaire-9 (PHQ-9): estimated completion time, 1 min			
	Self-reported daily condition: estimated completion time, 1 min	Daily at the end of work (not required)

(A) Collection of background factors

After obtaining written consent, the following information will be obtained from each participant:

(1) Sex, age, job department, job content, duration of service, position/title, family composition, work commute, household income, etc.;
(2) Information on past medical checkups and stress check information (with consent of participant);
(3) Any current illnesses and prescriptions.

(B) Collection of biological data with sensing devices

Biological information will be recorded at participants' workplaces during the 4-week observation period using methods B-1 through B-3, as described below:

(B-1) Pulse wave data

Participants install software on their work computers that uses a camera built into or connected to the computer to record video images of the participant; the pulse wave data is extracted from the facial video images. The pulse wave data is automatically sent to cloud storage through the software. Participants are asked to start the software when they arrive for work; the software must also be restarted if the participant's computer is put into sleep mode. This contactless pulse wave sensing system has a strong correlation in the R-R interval values compared to data obtained using ECG (*r*^2^ = 0.978, *p* < 0.00001) (19).

(B-2) Voice data

Participants install software on their work computers that uses a microphone built into or connected to their work computer to record the emotional components (pitch, speed, etc.) of participants' speech data. The emotional component data is automatically sent to cloud storage through the software. Participants are asked to start the software when they arrive for work; the software must also be restarted if the participant's computer is put into sleep mode.

(B-3) Electrodermal activity data, body motion data, and body temperature

Participants are asked to wear the Embrace2 wristband-type wearable device, made by Empatica, Inc., continuously during work hours. The device is equipped with an electrodermameter, accelerometer, gyroscope, and thermometer (20, 21).

(C) Collection of stress, well-being, and depression rating scale assessment data; self-reported daily condition

Researchers will send participants an email with a unique URL link for a unique website where participants can answer questionnaires on stress and well-being online. The evaluation scales and their estimated completion times are as follows (see Table 1 for the evaluation schedule):

- New Occupational Stress Questionnaire (modified version) (22)
- Perceived Stress Scale (PSS) (23)
- Satisfaction With Life Scale (SWLS) (24)
- Japanese version of Positive and Negative Affect Schedule (PANAS) (25)
- Japanese Flourishing Scale (FS-J) (26)
- Subjective Well-being/Ideal Happiness (27, 28)
- Japanese version of Patient Health Questionnaire-9 (PHQ-9) (29)
- Self-reported daily condition: an email with a unique URL will be sent to participants every business day; participants will input their condition (stress level, emotions, etc.) and sleep quality for the day in Likert scales, and include any special notes in 1–2 sentences (e.g., “I had a tough day,” “I was praised by my boss today,” etc.).

### Data Storage

The data of background factors (Data A) will be recorded on paper by the researcher and then entered by the researcher into the password-locked computer in the laboratory of the researcher and stored.

Pulse wave and voice data (Data B-1 and B-2) will be quantified and automatically uploaded to the server used by the researcher team through a Secure Sockets Layer (SSL) connection. The research team will download this data via an SSL connection to a research computer in the laboratory and analyzes it.

Data of electrodermal activity, body motion, and body temperature (Data B-3) will be captured by the wearable device and transferred to Empatica's cloud in encrypted form using proprietary software, where the raw data from the device will be analyzed and transformed into skin potential, heart rate, body movement, and temperature data. These sensing data will be eventually downloaded to the computers of the Keio University research team for analysis via an SSL connection.

The web-input data (Data C) such as stress, well-being, depression rating scale assessment data, and self-reported daily condition will be entered directly by participants by accessing an input format on a secure cloud computing service created by the research team. The entered data will be stored on the cloud, and the research team will access the cloud and downloads them to the laboratory's computer through an SSL connection.

All data above will be not accessible by the participants' employers.

### Data Analysis

The analysis workflow is as follows: (1) primary analysis, comprised of using software to digitalize participants' vital information; (2) secondary analysis, comprised of examining the relationship between the quantified vital data from (1), stress, well-being, and depression; (3) tertiary analysis, comprised of generating machine learning algorithms to estimate stress, well-being, and degree of depression in relation to each set of vital data as well as multimodal vital data. The primary analysis is conducted with technology already established by Panasonic and NEC, who are industrial collaborators in this study. In this research, the results of the primary analysis are used to generate machine learning algorithms for the secondary and tertiary analyses.

### Primary Analysis

#### Primary Analysis of Pulse Wave Data

Software from Panasonic installed on participants' work computers uses a camera to capture facial images of participants using facial detection. Based on skin color changes from blood flow in the images, the software extracts pulse wave data for the participant. The pulse wave data will then be clarified using filtering and noise removal techniques. SDNN, root mean square of successive differences in R-R intervals (RMSSD), Lorentz plot (Longitudinal axis/Transverse axis value), LH/HF ratio, and Tone-Entropy are calculated from the facial video images (1/30 s unit).

#### Primary Analysis of Voice

Software from Panasonic installed on participants' work computers uses a microphone to acquire speech data from participants. From this data, voice activity detection (VAD; presence/absence of voice), power (volume of speech), pitch, tension (strength of speech), and speech rate data are extracted (in units of 0.5 s), and an emotion estimate is calculated based on the results.

#### Primary Analysis of Electrodermal Activity

The Embrace2 wristband-type wearable device from Empatica, Inc., is equipped with an electrodermameter, accelerometer, gyroscope, and thermometer, which are used to record and analyze electrodermal activity, acceleration, angular velocity, and skin temperature.

### Secondary Analysis

Each result from the primary analyses of pulse wave, speech, and electrodermal activity data will be compared with the self-assessment results for stress, well-being, and depression. Then, we will determine the relationships between the vital data, stress, well-being, and depression. For example, such an investigation could be done by comparing the stress and well-being score quartiles with the vital data, or comparing them among subjects grouped according to depression symptom severity. Multiple regression analysis will be performed in order to predict stress, well-being, or depression using various kinds of vital data. Moreover, cluster analysis will be performed to find a group of people that have similar digital phenotypes and to seek for the potential relationships with clinical phenotypes.

### Tertiary Analysis

We will attempt to build a machine learning model to predict depression, stress, or well-being based on single modalities. Various methods, such as support vector machine, decision tree, and deep learning, are used for the machine learning analysis. Machine learning algorithms that estimate stress, well-being, and degree of depression are generated from each of these vital data sets. We will attempt to build a model not only for single modalities, but also one that can utilize all the modalities, namely pulse wave data, voice data, electrodermal activity data, body motion data, and body temperature data, together.

### Sample Size

**Figure 1:**
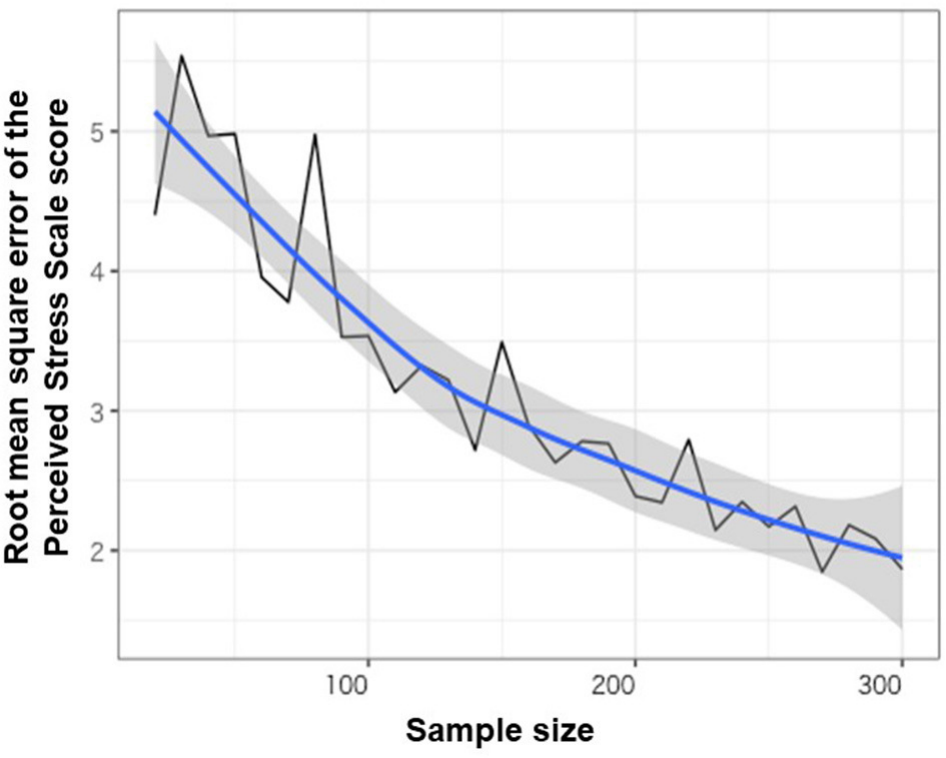
Sample size and root mean square error of the PSS score in the pilot study.

Based on the data obtained from the pilot study (28 cases) conducted prior to this study, we estimated the PSS scores using machine learning analysis of pulse wave and speech data. A gradient boosting decision tree was used for the machine learning algorithm, and hyper parameter was adapted by random search. Accuracy verification is based on 3-fold cross validation. In order to examine the accuracy for each data size, an arbitrary number of data points were extracted at random, and the accuracy for the data size was calculated. The pilot study sample size of 28 subjects was divided and incremented for prediction. The error in the PSS score range of 0–40 corresponds to a score of 2 when an error in the predicted value of PSS of up to around 5% is warranted. When aiming for a root mean square error of 2 or less, we found that 200 cases are required, as shown in Figure 1. Regarding dropouts, we considered that the 4-week-long observation as well as filling out multiple self-rating scales can be burdensome to participants, and the dropout rate can be high. Considering the dropout rate would be 30–35%, we will aim to recruit 300 cases.

## Discussion

The main aim of this study is to develop a technique to quantify stress and well-being in a way that does not disturb the workplace using vital data. Depression is a major problem in the field of occupational health, with its high incidence, especially among people of working age. The social significance of our project will be great because the occurrence of psychiatric disorders such as depression may be suppressed through self-management and improvement of working conditions in the companies if the quantitative measurement of stress and well-being, which is the ultimate goal of this study, can be achieved.

In this study, biometric information such as pulse wave, voice, and skin potential will be collected without any special intervention while the research collaborators are working. In addition, the research collaborators will be asked to cooperate in data entry through questionnaires, but as described below, the burden will be kept to a certain extent. We will use a web camera, a microphone, a wrist-band wearable device to obtain pulse, voice, and skin potential data, and a web-based questionnaire, which may cause psychological, physical, and time burdens. However, we believe that the burden will be small because we will not intervene and the questionnaire will be administered in a way that will not interfere with normal work and it takes a maximum of about 10 min per session.

The study will also include a rating scale for depression, on which significantly higher scores are presumed to indicate a higher risk of depression. If a recruited person will present any psychiatric disorder during the study, he or she will receive information that would not have been available to him or her if he or she had not participated in the study. The possibility that this may lead to a psychological burden on the individual cannot be denied. Only individuals who agreed that he or she will be informed of accidental findings are recruited. In addition, when the score of the rating scale for depression indicates he or she may have depression, an appropriate action should be taken, such as referring the participant to an appropriate medical institution if he or she wishes to do so. Moreover, the participant will be informed that sensing can be discontinued by shutting down the software or removing the wearable device, and that he or she may do so temporarily if it causes a psychological burden. Prior to the study, consent with the field companies will be obtained using a memorandum of understanding to ensure the privacy of research collaborators (e.g., not to disclose data without the participant's consent).

Challenges of the study are as follows. First, there will be a potential bias that those who are originally interested in the physical and mental health conditions may collaborate in the study. For this reason, participants who are currently being treated for psychiatric disorders, such as depression, will be excluded from the study, and efforts will be made to recruit a wide range of participants. Second, there will be also the issue of adherence. Failure to fill out the questionnaire or not obtaining biometric data may be considered. The researchers will try to reduce the burden on research participants as much as possible to minimize the time and effort required for inputting information and improve adherence. The researchers also consider rewarding each participant based on the response rate to the survey and the amount of time the participant will spend using the devices. Third, as the data collection is done in a natural setting in workplaces, we will lack the control data such as setting participants an experimental task where we can compare the data under strong stress.

## Ethics Statement

Our study has received approval from the institutional review board at Keio University School of Medicine. Approval was granted on April 22, 2019. This study is registered in the University Hospital Medical Information Network (UMIN) (UMIN000036814). Participants' inclusion will be voluntary. Written consent will be obtained from every participant. Participants will be free to withdraw from the study at any time.

## Author Contributions

KI, KM, and TK conceived the original study concept. KI designed and managed the study, and wrote the initial draft of the manuscript. KM and KS designed and managed the study. TK designed and supervised the study, and assisted in drafting the manuscript. All authors contributed to the design of the study, protocol development, its implementation, critically reviewed the manuscript, approved the final version of the manuscript, agree to be responsible for the accuracy, and integrity of the work.

## Conflict of Interest

The authors declare that the research was conducted in the absence of any commercial or financial relationships that could be construed as a potential conflict of interest.
